# Are Clean Eating Blogs a Source of Healthy Recipes? A Comparative Study of the Nutrient Composition of Foods with and without Clean Eating Claims

**DOI:** 10.3390/nu10101440

**Published:** 2018-10-05

**Authors:** Kacie M. Dickinson, Michelle S. Watson, Ivanka Prichard

**Affiliations:** 1 Nutrition and Dietetics, College of Nursing and Health Sciences, Flinders University, G.P.O. Box 2100, Adelaide, SA 5001, Australia; michelle.watson@flinders.edu.au; 2 Health and Exercise Science, College of Nursing and Health Sciences, Flinders University, G.P.O. Box 2100, Adelaide, SA 5001, Australia; ivanka.prichard@flinders.edu.au; 3 SHAPE Research Centre, Flinders University, G.P.O. Box 2100, Adelaide, SA 5001, Australia; 4 Flinders Centre for Innovation in Cancer, Flinders Drive, Bedford Park, SA 5042, Australia

**Keywords:** clean eating, nutrition, recipe, diet, food, internet, social media, blog, orthorexia, content analysis

## Abstract

Food blogs are an increasingly popular source of information about food and nutrition. There is a perception that foods published on clean eating blogs, which promote unprocessed foods, are healthier than comparable foods without these claims. However, foods with these claims and their nutrient composition have not previously been evaluated. The purpose of the study was to describe the nutritional content of clean eating recipes compared to recipes without clean eating claims and the nutritional guidelines published by the World Health Organisation (WHO). Clean eating recipes were systematically selected from 13 popular clean eating blogs and were described and compared with control recipes without clean eating claims. The nutrient profiles from the included recipes were summarised and evaluated against criteria from WHO recommendations for chronic disease prevention and criteria from the U.K. Food Standards Agency. Data for 86 clean eating recipes were extracted that represented five food categories: breakfast, snacks, treats, desserts, and smoothies. These were matched with 86 control recipes without clean eating claims. The clean eating recipes, per portion, provide the equivalent of 15% of daily energy intake. The average serving sizes were not significantly different between clean eating and control recipes. Overall, the clean eating recipes contained significantly more protein (8.1 ± 7.3 g vs. 5.7 ± 4.1 g, *p* = 0.01), fat (15.8 ± 10.6 g vs. 12.4 ± 9.3 g, *p* = 0.03), and fibre (5.0 ± 4.3 g vs. 2.8 ± 2.9 g, *p <* 0.01) per serving than control recipes. There were no significant differences between clean eating and control recipes with respect to the energy (1280 ± 714 kJ vs. 1137 ± 600 kJ, *p* = 0.16), carbohydrate (31.5 ± 27.3 g vs. 33.9 ± 19.4 g, *p* = 0.51), sugar (21.1 ± 20.9 g vs. 23.2 ± 14.9 g, *p* = 0.46), and sodium content (196.7 ± 269 vs. 155.8 ± 160.8, *p* = 0.23). Less than 10% of clean eating and control recipes met the WHO constraints for proportions of energy from fat and sugar intake. A simulated nutrient profile of an average clean and control recipe shows that nutrients for both are similarly classified as moderate to high in fat, saturated fat, salt, and sugar. Foods with clean eating claims contained the same amount of energy, sugar, and sodium as foods without those claims. Clean eating claims are potentially misleading for consumers who may believe these foods are healthy alternatives, potentially undermining people’s efforts to eat a healthy diet.

## 1. Introduction

In recent years, millions of people have turned to the Internet as a rapidly accessible source of health information, including information about diet. Websites and blogs devoted to food and nutrition have become increasingly popular, especially blogs promoting clean eating. Clean eating can be defined as choosing whole and minimally processed foods, and limiting consumption of artificial or processed foods [1]. Clean eating practices are centred around eating foods perceived as healthy. This often leads to highly restrictive diet practices. A strict adherence to eating patterns that focus on perceptions of “proper” nutrition is also encouraged [1,2].

Clean eating blogs are not usually authored by people qualified to give individual nutrition advice, with the majority of bloggers writing because of their passion for food [3,4]. Despite their popularity, it is suspected that in some cases, these clean eating blogs and the nutritional profile of the recipes they promote may not always be consistent with recommendations for good health and may portray inaccurate messages about food and nutrition, potentially reinforcing disordered eating behaviours in susceptible individuals [1,2,5].

No previous research has investigated the influence that this online information may have on people’s understanding about the healthfulness of foods. Ho and Chang Chien [6] found that attractive blog content conveyed a strong message of trustworthiness regarding the information conveyed and that the more a blog was considered trustworthy, the greater the likelihood the user would consume foods mentioned or recommended on it. This provides an indication of the subtle power bloggers possess and the potential for unintended health consequences from promoting certain types of foods or eating patterns. The ubiquitous nature and popularity of blogs raises concern over the content and impact of information presented by authors, especially with respect to dietary advice [7,8].

There are also concerns regarding the scientific validity and safety of some of the nutrition advice found on social media content, particularly regarding the encouragement of dietary restraint and restrictive eating patterns that focus on the elimination of many foods [8,9]. The intense focus on the nutritional quality of foods and extreme dietary restrictions have been implicated in orthorexia nervosa, defined as an extreme obsession with healthy eating, which has been reported to result in malnutrition and impaired social functioning [10,11,12]. Despite the serious impact some of this information might have, especially among vulnerable groups, there has not previously been any evaluation of the nutritional profile of the types of foods being promoted by bloggers, as well as that of comparable foods without claims.

There has only been one other analysis of the nutritional content of recipes published on food blogs, but none to date have looked at clean eating blogs. The authors found that the recipes on popular food blogs exceeded recommendations for daily sodium and saturated fat intake [4]. There has been one other study that analysed the content of popular websites used to access nutrition information. It found that commercial websites were used most of the time to access nutritional information and that most information found on these websites was inconsistent with national dietary guidelines [13].

Evaluation of the recipes presented on clean eating blogs is important for a number of reasons. Clean eating emphasises restrictive eating practices of varying intensity. In some cases, extreme dietary restrictions may adversely affect an individuals’ nutrient and energy intakes over time, especially among vulnerable groups [12]. No previous studies have focused specifically on the nutritional analysis of foods promoted on clean eating blogs, suggesting an important gap in the literature that requires further investigation.

To address this gap, we evaluated the nutritional content of foods from clean eating blogs online and compared them to the nutritional content of foods without clean eating claims, using recommendations for chronic disease prevention published by the World Health Organisation (WHO) [14]. We also used criteria from the U.K. Food Standards Agency (FSA) for front-of-pack labelling, as this is designed to guide consumers in selecting healthier foods [15]. We hypothesised that there would be no difference in the nutrient content of clean eating recipes compared with the recipes without these claims and that the clean eating recipes would exceed thresholds for nutrient content as defined by the WHO and the U.K. FSA.

## 2. Materials and Methods

### 2.1. Selection of Clean Eating Blogs and Recipes

**Figure 1:**
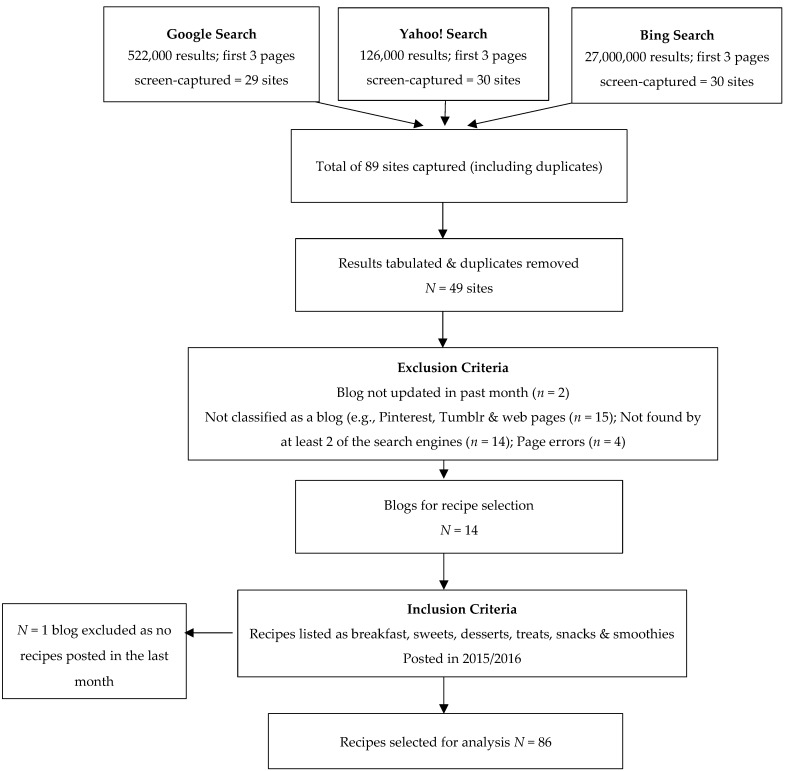
Flowchart of the process of blog and recipe selection.

We performed a cross-sectional content analysis to examine the nutritional values of recipes found on popular clean eating blogs. Following the search methodology used previously by Boepple and Thompson [16], web searches using Google, Yahoo!, and Bing with the keywords “clean eating”, “eat clean”, “blog”, “clean”, “eating”, and “eat”, were conducted in January 2016. Search engines such as Google were used because their search algorithms determine the ranking of the results based on how often the search term appears on the page, as well as the number of links to the page. This inspires confidence that the search results that were produced were ordered according to those most accessed and most referred to by other sites [17]. The first three pages of each search engine result were screen-captured, giving a total of 89 sites including duplicates. The results of the three pages of screen-captures were tabulated and any duplicated sites were removed. As blogs were the focus of this content analysis, web pages, Pinterest, and Tumblr sites were excluded. Uniform Resource Locators (URLs) that produced an error, blogs that had not been updated in the month prior to the search, and any blogs that had not been found by two of the three search engines were also excluded, leaving a total of 14 blogs for recipe selection. During the process of recipe selection, it was noticed that one of the blogs did not meet our blog selection criteria and it was therefore excluded, leaving 13 blogs for recipe selection and analysis (Figure 1).

Up to eight recipes were selected from each of the final 13 blogs. These met the criteria of being classified as breakfast recipes, snacks, smoothies, desserts, and treats and were posted within the previous 12 months. The categories selected represented the most popular types of recipes for foods promoted on clean eating sites. We did not consider other main meal categories or recipes as these were not a common feature or category of foods published on clean eating blogs. Any recipes for special occasions, e.g., birthdays or holidays such as Christmas and Easter, were excluded.

### 2.2. Selection of Control Recipes without Clean Eating Claims

A separate systematic search was conducted using Google to match and select control recipes for analysis based on recipe names within each food category. Using the name of the clean eating recipe, we conducted a search using Google with the keywords in the recipe as search terms. For example, for a clean recipe labelled “raw chocolate cheesecake”, the search terms, “chocolate cheesecake recipe” were used. The first page of the results was captured and the recipes were selected if they were the highest-ranking recipe returned on the first page of results. Recipes were screened for eligibility and included if they did not describe a diet or food philosophy on the blog or website they were published on (for example, healthy recipes, paleo, sugar-free). Overall, 86 recipes were matched across the five categories of recipes.

### 2.3. Nutritional Content of Included Recipes

A total of 172 recipes were included for analysis (86 clean eating recipes and 86 control recipes). For each recipe, information was collected about the type and quantity of ingredients and serving sizes. The information was entered into FoodWorks (Version 8, Xyris Software Pty Ltd., Spring Hill, QLD, Australia) for analysis. Foods were standardised as much as possible when entering the recipes to ensure similarity across the recipes for the nutrition analysis and comparison. For example, peanut butter was listed in recipes under several different names, so the same brand name was entered into FoodWorks. Proprietary foods and foods not found in FoodWorks databases, such as a brand name protein powders, had nutrient information imputed using the branded nutrient content information published online. The recipes were rechecked (by M.S.W.) after entry to ensure accuracy.

### 2.4. Statistical Analysis

For each recipe, we calculated the nutritional content per serving by dividing the total recipe by the number of servings described in the recipes. We used independent *t*-tests to compare the difference in nutrient content per serving for each recipe. Using Chi-squared tests, we compared the percentage contribution to daily energy and other key nutrients such as sugar, total fat, saturated fat, and sodium per serving, referring to guidelines published by the WHO for chronic disease prevention [14]. For each recipe, we assigned the macronutrient content (fat, saturated fat, sugar, and salt) a traffic light colour according to the nutrient profiling model proposed by the U.K. Food Standards Agency [15]. This system evaluates energy, fat, salt, sugar, and saturated fat per portion and per 100 g. To convert to grams of salt (NaCl), we multiplied sodium values by 2.5. Data are presented as *n* or (%) for frequencies and mean ± standard deviation where normally distributed and *p* < 0.05 was considered statistically significant. Statistical analyses were conducted using IBM SPSS version 22 (IBM Corporation, Chicago, IL, USA).

## 3. Results

A total of 172 recipes were selected for analysis across five product categories. The majority of the clean eating blogs was authored by one woman only (*n* = 10), one was authored by a male and female couple, and two blogs were authored by multiple people. The blogs were predominantly from the United States (*n* = 9), followed by Australia (*n* = 1), and the U.K. (*n* = 1). Geographic locations could not be determined for two of the blogs. Of the 86 clean recipes selected for analysis, 12.8% were classified as breakfast recipes (*n* = 11), 12.8% as desserts (*n* = 11), 29.1% as treats (*n* = 25), 33.7% as snacks (*n* = 29), and 11.6% as smoothies (*n* = 10). A comparison of the most frequent ingredients used in the clean and control recipes is reported in Table S1.

### 3.1. Nutrient Content of Clean Eating Recipes According to Food Category

The nutrient content of the recipes published on the clean eating blogs according to our food categories is reported in Table 1. The categories of recipes were: breakfast recipes, snacks, smoothies, desserts, and treats. The mean serving size of the clean eating recipes we evaluated was 175.7 ± 195.3 g. The dessert category contained the highest amount of energy per serving (1583 ± 651 kJ), equating to 18% of an individual’s recommended daily kilojoule intake. From highest to lowest contributor to energy intake, the categories were then: breakfast recipes, smoothies, treats, and finally snacks. In contrast, energy density levels were highest for treats and snacks (13.1–16.5 kJ/g).

### 3.2. Comparison of Clean Eating Recipes with Control Recipes without Clean Eating Claims

Overall, there was no significant difference in serving size between clean eating and control recipes (Table 2). Per serving, the clean eating foods contained the same amount of energy, carbohydrate, sugar, and sodium as the control recipes. However, the clean eating recipes contained significantly more protein, fat, and fibre per serving than controls (Table 2). In addition, there was a significant difference in carbohydrate and sugar content per 100 g (Table 2).

### 3.3. Evaluation of Foods Using WHO Guidelines for Chronic Disease Prevention and FSA Traffic Light Criteria

Table 3 presents the proportion of analysed recipes that were within the recommendations published by the WHO for nutrient intake for chronic disease prevention [14]. Overall, a low proportion of the clean eating and control recipes met recommendations for fat and sugar intake for chronic disease prevention. There was a significant difference between the proportion of recipes that met recommendations for energy from carbohydrate (15% clean eating recipes vs. 37% of control recipes) and energy from monounsaturated fat (27% vs. 48% clean and control recipes respectively, Table 3).

We also applied the U.K. Food Standards Agency’s criteria [15] for front-of-pack labelling to each recipe. The number of recipes that would qualify for red, amber, or green categories for these nutrients is summarised in Table 4. Overall, recipes most frequently met acceptable criteria (green criteria; “low in”) for salt, followed by fat, and then sugar. There were fewer clean eating recipes that met acceptable criteria for sugar and total fat, compared with control recipes. The average nutrient profile for clean and control recipes is displayed in Table 5. Overall, the profile for the macronutrients is very similar for the two categories of recipes. Total fat and salt were similarly classified as moderate (amber colour), and saturated fat was classified as high (red) for both clean and control recipes. Average sugar content was moderate for clean recipes (amber) and high (red) for control recipes, although the absolute mean difference in sugar content was small (21.1 g vs. 23.2 g per portion clean and control recipes, respectively).

## 4. Discussion

This is the first comprehensive description of the nutrient composition of foods published on clean eating blogs. The results demonstrate that the recipes promoted on clean eating blogs had overall similar energy and nutrient profiles compared with those of similar foods without clean eating claims. The main differences observed in nutrient content were that the clean eating recipes were higher in protein, fat, and fibre. However, the overall nutritional quality of all of the products included in the analyses was poor, with very high amounts of sugar, total fat, and saturated fat. Few recipes met the WHO guidelines for limiting sugar and fat intake to prevent chronic disease [14]. This was also reflected in the evaluation of recipes against U.K. FSA criteria, demonstrating “moderate to high” in ratings for sugar, fat, and salt.

Previous research evaluating the nutritional profile of recipes published online is limited and no studies have previously looked at foods promoted on clean eating blogs. Schneider et al. [4] previously evaluated six popular food blogs and found that recipes published on these blogs, across all recipe categories, had excessive fat and sodium content, but that their energy content was not excessive. Other research has evaluated recipes in cookbooks published by celebrity chefs. These were also found to be of poor nutritional quality, with many exceeding thresholds for sugar, fat, and sodium content [18]. In similar research, Trattner et al. [19] examined the healthiness of 5327 main meal recipes from the Internet by evaluating the proportion that met all of the WHO’s criteria. The findings were that Internet recipes were less healthy than TV chef and pre-prepared ready meals in the U.K., with only six recipes overall (0.001%) meeting the WHO criteria for chronic disease prevention [14]. In the present study, the number of recipes meeting the WHO criteria was still relatively low and the proportion of clean eating recipes meeting guidelines was significantly lower than that of control recipes for monounsaturated fat and carbohydrate. This reflects the overall poor nutritional quality of the recipes we sampled, which were moderate in salt content and high in saturated fat. The overall poor nutritional profile of the recipes we evaluated is concerning given the volume and growing influence of food and nutrition information published online. Consumers are increasingly looking to bloggers and celebrities as sources of nutritional information [20], so this warrants concern when this information may be disseminated by individuals without qualifications who might not be communicating evidence-based or even correct information to consumers.

There has been a large increase in the popularity of eating “clean”, driven by the perception that such types of foods are healthier. However, we observed no significant differences in energy, sugar, or sodium content between clean eating recipes and control recipes. This is likely because many substitute ingredients promoted in clean eating recipes have either similar caloric value or nutritional value to regular ingredients that would be used in these types of recipes. For example, we observed the use of products such as coconut oil, maple syrup, and nut milks in the clean recipes instead of butter, sugar, and cow’s milk that feature in the control recipes (Table S1).

However, we also found clean eating recipes to be significantly higher in protein, fat, and fibre per serving. This is likely explained by the frequency of higher protein ingredients such as nuts, eggs, protein powder, and nut-based flours (almond meal) among the clean eating recipes. The higher protein and fibre content of the clean recipes may have implications for appetite and body weight. In epidemiologic studies, higher fibre intakes are associated with lower body weight [21]; however, a systematic review and meta-analysis of fibre supplementation studies demonstrated that fibre does not have a short term impact on food intake or appetite [22].

We speculate that consumers may infer health benefits about foods labelled as “clean” because of a “halo” effect. This refers to a consumer tendency of inferring health benefits above and beyond attributes that the claim communicates [23]. The halo effect of nutrition claims has previously been reported in literature and findings support the view that claims about foods can shift people’s attitudes towards food and even influence food consumption, such that they consume up to 35% more of a food if it is regarded as a healthier version of the same product [24,25].

As clean eaters tend to fixate on the techniques and ingredients used in food preparation, consumers may perceive foods in their more “natural” state to be healthier and to contain fewer calories than the same food that has been processed mechanically. This was evaluated in a series of studies by Szocs et al. [26], who tested the effect of processing (juicing and blending) test foods on consumer perceptions of their caloric content and healthfulness. They found that consumers perceived the more processed foods as less healthy and containing more calories, despite the volume of food being kept constant. Clean eating may also have the same effects on attitudes and consumption but this has not previously been tested in an experimental setting. These results highlight the need to conduct further research in this area about people’s perceptions of these types of foods and on the impact on food choice and consumption behaviours.

One of the strengths of this study was the systematic process used to select popular clean eating blogs. The sampling method was based on previous methods used for the content analysis of healthy living blogs [16]. We sampled recipes across five different food product categories. Breakfast was the only meal covered (we did not sample lunch and dinner as they are not common categories of food published on clean eating blogs). However, the inclusion of lunch and dinner recipes may reveal different results and is something for consideration in future research.

It is important to note that the we evaluated the nutrient composition of recipes consumed as a single food, not that of meals or the daily intake of food of an individual, which would comprise combinations of multiple foods. Therefore, the comparison of the recipes according to the percentage of energy from macronutrients should be viewed with caution. Depending on what other foods an individual usually consumes over the course of a day, their overall intake may fall within these constraints.

We did not impose any geographic restrictions on the blogs eligible for inclusion in the study. However, the final sample featured blogs published mainly in the U.S.A., so findings may not be generalisable to other countries. The average serving sizes within a food product category did not significantly differ, so it was appropriate that the nutrient content of the recipes was comparable per portion consumed.

This research provides important information about the health and nutrition information landscape on social media and the web, which is an increasingly popular avenue the general public are turning to for advice. It can inform practitioners and policy-makers about some of the messages on social media and some of the information being circulated. In turn, this can inform health promotion strategies for communicating messages about food and nutrition. This research is useful to clinicians trying to understand the healthfulness of popular health and nutrition content online so they can better inform and educate their patients about the quality of the information available. It also highlights an opportunity for health professionals to harness the influence of social media/online space to provide credible nutrition information. There may be some lessons to learn from bloggers on how health professionals can effectively communicate with consumers as trusted sources of information. 

## 5. Conclusions

The clean eating recipes evaluated had a small, but significantly higher protein, fat, and fibre content than foods not published on clean eating blogs, but were similar in overall energy and sugar content. As such, following clean eating recipes is unlikely to confer any additional health benefits to the average person. The consumption of processed and discretionary foods needs to decrease at a population level and clean eating alternatives promoted online may not represent healthier alternatives.

## Figures and Tables

**Table 1:** Descriptive analysis of recipes from clean eating blogs.

Nutrients per Serving	Recipe Category											
	Total (*n* = 86)		Breakfast (*n* = 11)		Snacks (*n* = 29)		Smoothies (*n* = 10)		Desserts (*n* = 11)		Treats (*n* = 25)	
	Mean	SD	Mean	SD	Mean	SD	Mean	SD	Mean	SD	Mean	SD
Serving Weight (g)	175.7	195.3	257.2	134.7	90.9	75.0	526.0	326.5	193.4	93.1	90.2	82.0
Energy (kJ)	1280.2	714.8	1521.3	505.7	1089.4	740.0	1489.0	693.1	1582.6	651.1	1178.7	752.3
Energy density (kJ/g)	11.8	6.4	13.1	4.0	3.2	1.4	9.3	4.5	16.5	6.4	7.9	4.9
Protein (kJ)	8.1	7.3	11.1	5.3	7.2	5.5	13.8	13.7	6.6	3.7	6.2	6.5
Energy from protein (%)	10.4	7.3	13.3	8.2	11.2	6.5	15.0	12.0	6.8	2.7	8.1	5.1
Total fat (g)	15.8	10.6	16.2	6.1	12.7	7.2	10.7	10.0	22.5	13.7	18.2	12.6
Energy from fat (%)	46.5	21.5	41.4	15.2	46.3	20.5	26.0	23.2	48.8	23.5	56.1	18.5
Saturated fat (g)	6.4	6.4	7.9	6.0	5.1	4.3	2.0	2.0	8.6	7.9	8.2	7.9
Polyunsaturated fat (g)	2.8	2.5	2.6	1.8	2.8	2.1	2.7	2.9	3.9	3.7	2.3	2.4
Monounsaturated fat (g)	5.5	5.0	4.4	2.7	4.0	3.0	5.2	5.8	8.5	7.1	6.5	5.7
Carbohydrate (g)	31.5	27.3	40.8	20.7	28.2	29.8	47.7	43.0	36.1	21.5	22.6	16.8
Energy from carbohydrate (%)	38.1	20.8	37.4	19.1	39.2	21.0	49.5	25.0	40.7	24.6	31.4	16.4
Sugar (g)	21.1	20.9	17.8	12.5	16.7	14.2	42.0	41.2	28.9	19.5	15.9	13.5
Dietary fibre (g)	5.0	4.3	6.5	3.1	4.3	4.0	8.1	3.6	5.9	5.4	3.6	4.2
196.7	269.0	115.2	87.6	295.0	305.3	128.3	129.2	134.3	147.1	173.2	331.8

**Table 2:** Comparison between clean eating recipes and controls per serving and per 100 g.

Nutrient	Per Serving						Per 100 g					
	Clean		Control		Mean Diff	*p*	Clean		Control		Mean Diff	*p*
	Mean	SD	Mean	SD			Mean	SD	Mean	SD		
Weight (g)	175.7	195.3	144.6	134.8	31.1	0.23						
Energy (kJ)	1280.2	714.8	1137.3	600.1	142.9	0.16	1176.6	640.0	1221.6	648.0	−45.0	0.65
Protein (kJ)	8.1	7.3	5.7	4.1	2.4	0.01	6.6	4.5	5.3	2.8	1.3	0.03
Total fat (g)	15.8	10.6	12.4	9.3	3.3	0.03	17.2	14.2	15.2	12.0	2.1	0.30
Saturated fat (g)	6.4	6.4	5.9	5.6	0.6	0.53	7.5	9.6	7.2	7.0	0.3	0.80
Polyunsaturated fat (g)	2.8	2.5	1.7	1.9	1.0	0.00	2.7	2.5	2.2	3.4	0.6	0.21
Monounsaturated fat (g)	5.5	5	4.0	3.4	1.5	0.02	5.9	5.8	4.8	5.1	1.1	0.21
Carbohydrate (g)	31.5	27.3	33.9	19.4	−2.4	0.51	24.1	14.3	33.6	16.6	−9.5	0.00
Sugar (g)	21.1	20.9	23.2	14.9	−2.0	0.46	16.1	11.3	22.5	13.9	−6.4	0.00
Dietary fibre (g)	5	4.3	2.8	2.9	2.2	0.00	4.0	2.8	2.4	1.9	1.5	0.00
Sodium (mg)	196.7	269	155.8	160.8	40.9	0.23	187.6	244.2	165.5	161.3	22.1	0.48

**Table 3:** Percentage of energy from macronutrients from clean eating recipes compared with controls.

Nutrient	Clean Eating Recipes		Control Recipes		WHO Recommendations	Chi Squared	*p* Value
	*n*	% within World Health Organisation (WHO) Range	*n*	% within WHO Range			
Protein	14	16	19	22	10–15%	0.937	0.333
Total Fat	4	5	6	7	15–30%	0.425	0.515
Polyunsaturated fat	20	23	15	17	6–10%	0.897	0.344
Monounsaturated fat	23	27	40	47	9–20%	7.239	0.007
Saturated fat	28	33	23	27	<10%	0.697	0.404
Carbohydrate	13	15	32	37	55–75%	10.865	0.001
Sugar	11	13	5	6	<10%	2.481	0.115

**Table 4:** Number of recipes that met the U.K. Food Standards Agency (FSA) nutrient profiling criteria.

Nutrient	No. for Clean Eating Recipes			No. for Control Recipes		
	Red	Amber	Green	Red	Amber	Green
Sugar	39	42	5	20	53	13
Fat	32	41	13	39	26	21
Saturated Fat	47	17	22	39	26	21
Salt	3	47	36	7	32	47

**Table 5:** Mock Traffic Light Label using FSA criteria for average nutrient values of recipes according to their clean or control status.

Recipe Type (Per Average Portion)	Energy	Fat (g)	Saturated Fat (g)	Sugar (g)	Salt (g)
Clean Eating Recipes	1280 kJ	15.8 ^a^	6.4 ^b^	21.1 ^a^	0.5 ^a^
*%RI*	15%	23% ^a^	32% ^b^	23% ^a^	8% ^a^
Control Recipes	1176 kJ	12.4 ^a^	5.9 ^b^	23.2 ^b^	0.4 ^a^
*%RI*	14%	18% ^a^	30% ^b^	26% ^b^	7% ^a^
*Note.*^a^ moderate (amber) rating, ^b^ high (red) rating %RI (Percent Reference Intake)					

## Supplementary Materials

The following are available online at http://www.mdpi.com/2072-6643/10/10/1440/s1: Table S1: Top ranking ingredients according to recipe category among clean and control recipes.
