# Supplementary material for: Are Clean Eating Blogs a Source of Healthy Recipes? A Comparative Study of the Nutrient Composition of Foods with and without Clean Eating Claims
Source: Nutrients. 2018 Oct 5;10(10):1440. doi: 10.3390/nu10101440 (PMC6213725; doi:10.3390/nu10101440)
Supplement: Supplementary file 1 [file nutrients-10-01440-s001.pdf]

Supplementary Table 1: Top ranking ingredients according to recipe category among clean and control recipes

Breakfast (n=11) Ingredient Name	n	% of recipes in category	Snacks (n=29) Ingredient Name	n	% of recipes in category	Smoothie (n=10) Ingredient Name	n	% of recipes in category	Dessert (n=11) Ingredient Name	n	% of recipes in category	Treat (n=25) Ingredient Name	n	% of recipes in category	Overall (n=86) Ingredient Name	n	% of recipes in category
Clean Recipes																	
Oats	7	64%	Egg	15	52%	Honey	4	40%	Vanilla extract	5	45%	Nuts	13	52%	Coconut oil	29	34%
Honey	5	45%	Baking powder	14	48%	Banana	3	30%	Cinnamon	4	36%	Chocolate	11	44%	Salt	27	31%
Coconut oil	4	36%	Salt	13	45%	Fresh berries	3	30%	Nuts	4	36%	Salt	11	44%	Honey	29	34%
Cinnamon	4	36%	Coconut oil	13	45%	Cucumber	3	30%	Cacao	3	27%	Honey	10	40%	Nuts	27	31%
Fresh berries	4	36%	Nuts	10	34%	Orange juice	2	20%	Maple syrup	3	27%	Coconut oil	10	40%	Egg	18	21%
Banana	4	36%	Oats	10	34%	Avocado	2	20%	Apple	3	27%	Maple syrup	8	32%	Oats	17	20%
Coconut milk	3	27%	Honey	10	34%	Spinach	2	20%	Coconut oil	2	18%	Cacao	7	28%	Chocolate	13	15%
Almond milk	3	27%	Spices	11	38%	Apple	2	20%	Chocolate	2	18%	Butter	7	28%	Coconut milk	11	13%
Eggs	3	27%	Vegetable oil	6	21%	Protein powder	2	20%	Coconut milk	2	18%	Coconut milk	6	24%	Maple syrup	11	13%
Salt	3	27%	Peanut butter	6	21%	Almond Milk	2	20%	Evaporated cane sugar juice	2	18%	Coconut sugar	6	24%	Cacao	10	12%
Control Recipes																	
Oats	8	73%	Flour	23	79%	Banana	7	70%	Butter	7	64%	Butter	17	68%	Butter	50	58%
Cow’s milk	7	64%	Butter	23	79%	Yoghurt	6	60%	White sugar	6	55%	Flour	12	48%	Flour	41	48%
Brown Sugar	6	55%	Egg	21	72%	Honey	6	60%	Brown sugar	6	55%	Brown sugar	12	48%	Brown Sugar	40	47%
Apple	5	45%	Brown Sugar	16	55%	Water	6	60%	Flour	6	55%	White sugar	11	44%	Eggs	37	43%
Cinnamon	4	36%	Baking powder	12	41%	Fresh berries	4	40%	Vanilla extract	5	45%	Chocolate	11	44%	White sugar	25	29%
Fresh berries	4	36%	Cows milk	11	38%	Cow’s milk	4	40%	Egg	5	45%	Eggs	11	44%	Cow's milk	22	26%
Banana	4	36%	Nuts	10	34%	Avocado	2	20%	Cream	5	45%	Nuts	10	40%	Nuts	20	23%
Salt	3	27%	White sugar	8	28%	Spinach	2	20%	Cocoa powder	5	45%	Vanilla extract	9	36%	Vanilla extract	14	16%
Honey	3	27%	Cinnamon	6	21%	Peach	2	20%	Apple	4	36%	Cocoa Powder	6	24%	Chocolate	15	17%
Butter	3	27%	Chocolate	4	14%	Nut butter	2	20%	Cinnamon	3	27%	Coconut	5	20%	Cinnamon	13	15%
